# Implementation status of the nationally harmonized competency-based, integrated, modular medical curriculum in Ethiopia: opportunities and challenges

**DOI:** 10.1186/s12909-024-05796-2

**Published:** 2024-07-25

**Authors:** Abinet Gebremickael, Abay Mulu

**Affiliations:** 1 Department of Anatomy, College of Medicine and Health Sciences, Arba Minch University, Arba Minch, Ethiopia; 2 Department of Anatomy, School of Medicine, College of Health Sciences, Addis Ababa University, Addis Ababa, Ethiopia

**Keywords:** Competency-based medical curriculum, Challenges, Ethiopia, Implementation, Opportunities

## Abstract

**Background:**

Well-designed curriculums are crucial for quality education. This study aimed to assess the implementation status of a harmonized competency-based medical curriculum (CBMC) in Ethiopia, as well as identify the associated challenges and benefits.

**Methods:**

A mixed-method approach was used. Data collection took place from September 1, 2023, to October 15, 2023. Eleven medical schools were randomly selected from a list of 28 public medical schools in Ethiopia. Participants were purposively chosen from selected medical schools using a controlled sampling method. A total of 121 participants took part in the survey. Interviews and focus group discussions were conducted with key informants to complement the quantitative findings. Descriptive statistics, such as frequencies and percentages, were used to summarize the quantitative survey responses. Thematic content analysis was used to analyze the qualitative data.

**Results:**

The implementation of a CBMC in Ethiopian public universities faces various challenges and provides opportunities. Around 22% of respondents mentioned that the whole group session (WGS) had never been implemented in their school. Regarding community-based learning (CBL), 64.6% of respondents noted that it was intermittently implemented in their institution. Only 32% of respondents considered students’ clinical exposure during preclerkship years to be reasonable. Interestingly, approximately 73% of respondents stated that problem-based learning (PBL) activities were regularly conducted in their school. Around 68% of respondents believed that competency-based assessment methods were moderately utilized. While many participants perceive the curriculum as having positive content alignment and structure with intended learning outcomes, challenges related to practical sessions, resource limitations, and uneven access to training opportunities persist. Resource constraints are a prominent challenge. The opportunities identified include early clinical exposure and enhanced communication skills.

**Conclusions:**

This survey highlights the need for ongoing curriculum evaluation and fine-tuning to ensure its success.

## Introduction

A competency-based curriculum (CBC) is an educational approach that focuses on developing specific competencies, skills, and knowledge required for real-world application in a particular field or profession. The origins of competency-based education can be traced back to the mid-20th century, when it emerged as a response to the need for more practical and outcome-based education [1, 2]. In the field of medical education, the transition to competency-based medical education was influenced by the need to address certain shortcomings of the traditional model. Medical educators and researchers recognize that medical training should not only emphasize knowledge acquisition but also develop skills in clinical reasoning, effective communication, and evidence-based decision-making [1, 3, 4].

There were several incomplete, outdated, and traditional medical curricula for undergraduate medical training in Ethiopia. The traditional/conventional curriculum was discipline-based, and the courses are fragmented in which different subjects were taught separately. Additionally, the teaching methodology consists mainly of didactic lectures and there is a loose connection between the world of education and the world of work [5, 6]. The findings of a national task analysis survey that assessed health professionals’ competency and graduate competency assessment revealed that physicians taught with the conventional curriculum do not obtain the competencies they require before entry into practice [5].

Understanding the shortfalls of traditional curriculum, the nationally harmonized competency-based modular medical curriculum (CBMC) was designed and introduced in Ethiopia since 2020. The CBMC is produced as an integrated modular approach in which the basic sciences are taught simultaneously with clinical and public health. Integration is done using body systems and thematic areas as an organizing framework. Each module is a set of well-defined topics and contents from basic sciences, clinical discipline, generic skills, and public health [Harmonized medical curriculum, 2021].

This CBMC is designed by incorporating innovative educational and assessment strategies. These include: (1) *Student-centered learning* (SCL), (2) *Problem-based learning* (PBL) - a method of learning in which learners first encounter a problem, in which cases are crafted in consideration of real-life situations, followed by a systematic, learner-centered inquiry and reflection process, (3) *Community Based Learning* (CBL) - include learning activities that take place outside academic hospital in the community setting, (4) *Whole group session* (WGS) - a session in which students consolidate and reflect on the different learning activities covered during the week, (5) *Objective structured clinical examination* (OSCE) - a performance-based exam in clinical setting.

One of the objectives of the harmonized curriculum is to prepare medical doctors who address the health priority needs of the Ethiopian population and its health system challenges [Harmonized medical curriculum, 2021]. Harmonization of medical curricula was also aimed at ensuring consistency in educational content, learning outcomes, teaching methods, and assessment approaches across different medical institutions in the country.

Related studies mainly advocate the benefits of competency-based medical education [7–12]. However, with the recent implementation of the harmonized CBMC in Ethiopia, it is crucial to evaluate the progress of different aspects of the curriculum and identify the challenges that come with its implementation. By understanding the perspectives of those responsible for implementing the curriculum, we can identify any gaps or challenges early on. This will provide us with valuable insights and recommendations for successful implementation, as well as support evidence-based decision-making for potential revisions to the curriculum. Consequently, the main objective of this study is to assess the implementation status of the key components of Ethiopia’s harmonized medical curriculum, while also examining the challenges and opportunities that arise.

## Methods

A mixed-methods approach involving both quantitative and qualitative data collection methods was utilized. The data collection process started with collecting and analyzing quantitative data. Subsequently, qualitative data was gathered to supplement the quantitative findings and gain a more in-depth understanding of specific variables that required deeper exploration from the quantitative survey. Building on the findings from the quantitative analysis, interview guiding questions were then developed to further explore these variables to ensure a comprehensive understanding. The data collection was conducted from September 1, 2023, to October 15, 2023, in selected public medical schools.

As the representativeness of the survey participants is crucial for the generalizability of the findings, efforts were made to ensure that the selected participants truly reflected the broader population of medical schools in Ethiopia. Accordingly, eleven medical schools were randomly selected for this survey from a list of 28 public medical schools in Ethiopia. These institutions are as follows: Arba Minch University, Hawassa University, Wolaita Sodo University, Wolkite University, Dire Dawa University, Mizan Tepi University, Debre Tabor University, Arsi University, Jimma University, Wachemo University, and Haramaya University.

Subsequently, participants for the survey questionnaire were purposively and nonrandomly chosen from each selected medical school using a controlled sampling method. As the curriculum was recently implemented, only courses for preclinical years have been completed in most schools. Therefore, participants were selected with the aim of ensuring their representation of a diverse field of preclinical specializations or courses taught and the inclusion of key academic leaders from medical schools. Given the impracticality of calculating a precise sample size, a total of 121 participants, comprising eight to thirteen individuals from each school, participated in the survey. This group included a variety of experts, such as anatomists, physiologists, biochemists, pharmacologists, microbiologists, pathologists, public health educators, physicians, school deans, preclinical or module coordinators, and quality assurance office coordinators. This approach was designed to capture a broad spectrum of perceptions and insights related to the curriculum.

Quantitative data were collected from faculty members and relevant academic leaders from the selected schools by using a structured survey questionnaire. The survey was structured into four key sections: the demographic and professional characteristics of survey participants, experiences with the implementation of various aspects of the curriculum, participants’ perceptions of the curriculum design and structure, and the opportunities and challenges associated with the implementation of a competency-based curriculum. The survey questionnaire was designed and administered through both the Kobo Toolbox, a digital data collection platform, and printed copies for the accessible target survey participants.

The survey tool was developed by conducting a comprehensive literature review on competency-based medical education and curriculum implementation. Additionally, the harmonized medical curriculum itself was also carefully reviewed. This review helped to identify key themes and variables relevant to the study. Input was also sought from faculty members and academic leaders with extensive experience in medical education. The draft survey was then pilot tested with a small, representative sample of participants from Worabe University, one of the medical schools not included in the main study. This pilot testing aimed to identify any issues with the survey questions, such as ambiguity or technical problems with the digital data collection tool (Kobo Toolbox). After making necessary adjustments, the survey was finalized and prepared for administration to the target participants. For the digital platform, target participants were invited to participate via multiple communication channels, including Telegram and email.

Qualitative data were collected through interviews and focus group discussions using a semi-structured interview guide that allowed for probing and follow-up questions. The interviews and FGD continued until information saturation was reached for each focus area, meaning that no new significant information emerged. This point was reached during the 5th FGD, that is why the study was limited to five schools. Accordingly, key informants who participated in this study were from five medical schools: Arba Minch University, Wolaita Sodo University, Hawassa University, Wachemo University, and Wolkite University. In each FGD, key informants were purposively selected from various roles within the medical schools, including faculty members who have direct engagement in the medical curriculum implementation, medicine school deans, preclinical or module coordinators, and quality assurance office coordinators.

### Statistical analysis

Descriptive statistics such as frequencies and percentages were computed to summarize and present the quantitative survey responses in different domains by using SPSS version 25 statistical software. For the qualitative data, the audio recordings of the interviews and FGDs in Amharic were transcribed to word text and then translated to English. After the data were repeatedly read, response patterns were identified, coded, categorized, and organized into themes. Since the focus areas for the FGD were limited and the responses obtained from participants were less diverse, the analysis did not involve the complex steps often performed with qualitative content analysis. Therefore, in this qualitative study, the key concepts of each theme were summarized and presented after performing simple thematic content analysis manually. Triangulation of findings from both types of data was performed to strengthen the discussion and overall conclusion of the study.

## Results

### Quantitative assessment findings

#### Demographic and professional characteristics of the survey participants

The majority of survey participants were male and between the ages of 25–34. Among the respondents, 39% held academic leadership positions. Only about 9% of respondents had a PhD. 53% of respondents held the rank of Lecturer, and the rest were Assistant Professors. The participants had diverse fields of specialization, mostly preclinical. Around 47% of respondents had over 10 years of teaching experience in higher education institutions (Table 1).

**Table 1:** Showing demographic and professional profiles of the survey participants in Ethiopian public medical schools, 2023

Variable	Category		Frequency	Percent
Gender		Male	108	89.26%
		Female	13	10.74%
Age Groups		25–34	85	70.25%
		35–44	36	29.75%
Positions		Academic leadership positions	47	38.84%
		Faculty	74	61.15%
Educational Qualifications		Master’s degree (MSc)	93	76.86%
		Doctoral degree (PhD)	11	9.10%
		Medical doctor (MD)	17	14.05%
Academic Ranks		Assistant Professor	57	47.10%
		Lecturer	64	52.89%
Field of Specializations		Anatomist	26	21.50%
		Physiologist	20	16.52%
		Biochemist	17	14.05%
		Microbiologist	16	13.22%
		Pathologist	12	9.92%
		Pharmacologist	14	11.57%
		Public health specialist	9	7.44%
		Medical Doctor (MD)	5	4.13%
		Other preclinical specialty	2	1.65%
Service Years in Higher Education Institutions after obtaining MSc degree		over 10 years of experience	57	47.10%
		4–6 years	28	23.14%
		1–3 years	9	7.44%
		7–9 years	23	19.00%
		less than 1 year	4	3.30%

#### Experiences in the implementation of the various aspects of the curriculum

Approximately 59% of respondents reported receiving orientation sessions or workshops to familiarize faculty with the new CBMC. About 60% of respondents attended relevant capacity-building training programs adequately. Only about 4% of respondents indicated that their institution had undertaken a reasonable curriculum review, in line with the curriculum’s standards, and customization to adopt the harmonized curriculum. Around 22% of respondents mentioned that WGS had never been implemented in their school, while others reported minimal to moderate implementation. Interestingly, approximately 73% of respondents stated that PBL activities were regularly conducted in their school. Regarding competency-based assessment methods, such as the OSCE, around 68% of respondents believed that they were moderately utilized in their school. When it came to the use of standardized assessment rubrics and checklists during competency-based assessments, 77.7% of respondents stated that these tools were partly developed or adopted and used in their institution. In terms of CBL, the majority (64.6%) of respondents noted that it was intermittently or occasionally implemented in their institution. Regarding students’ clinical exposure during preclerkship years, only 32% of respondents considered it reasonable, while 34% had limited exposure and 21% had no exposure (Table 2).

**Table 2:** Showing experiences in the implementation of various aspects of the harmonized competency-based medical curriculum in Ethiopian public medical schools, 2023

Variables	Response Category	Frequency	Percent
Organizing orientation workshops to the faculty on the new curriculum	Orientation workshop was not organized	37	30.6
	Orientation workshop was organized, but didn’t attend	13	10.7
	Yes, attended orientation workshop	71	58.7
	Total	121	100.0
Faculty’s attendance of capacity-building training sessions related to competency-based education	Not at all	2	1.7
	Yes, adequately	73	60.3
	Yes, but inadequately/partially	46	38.0
	Total	121	100.0
Implementation of whole group sessions (WGS)	Minimally implemented	37	30.6
	Moderately implemented	57	47.1
	Not implemented	27	22.3
	Total	121	100.0
Curriculum review and customization to adopt the harmonized curriculum	Yes, has been undertaken reasonably	5	4.1
	Limited review and modifications undertaken	46	38.0
	Moderate review and modifications undertaken	37	30.6
	No review conducted	33	27.3
	Total	121	100.0
Implementation of problem-based learning (PBL)	Intermittently or occasionally conducted	22	18.2
	Rarely conducted	11	9.1
	Regularly or routinely conducted	88	72.7
	Total	121	100.0
Utilization of competency-based assessment methods (OSCE)	Minimal utilized	22	18.2
	Moderate utilized	82	67.8
	Substantial utilized	17	14.0
	Total	121	100.0
Utilization of standardized assessment rubrics during competency-based assessments	Effectively developed/adopted and used	14	11.6
	Not developed/adopted or used	13	10.7
	Partly developed/adopted and used	94	77.7
	Total	121	100.0
Implementation of community-based learning	Consistently implemented	28	25.5
	Intermittently or occasionally implemented	71	64.6
	Not implemented	11	10.0
	Total	110	100.0
Preclerkship years clinical exposure (hospital visit)	A reasonable exposure	32	32.3
	Limited exposure	34	34.3
	Moderate exposure	12	12.1
	No exposure	21	21.2
	Total	99	100.0

#### Perceptions of faculty members regarding curriculum design and structure

According to the survey, the alignment of the curriculum design with the intended learning outcomes was perceived positively by 59% of the participants. In contrast, the other 41% considered it negative. In terms of the curriculums’ specific content and structure, approximately 76% of the participants found it conducive and highly conducive to effective teaching and learning. Conversely, approximately 24% of participants found it negative. With regard to the incorporation of practical sessions, only approximately 15% of the participants felt that they were adequately incorporated into the curriculum. Regarding comfort with the teaching methodologies used in the curriculum, over half of the respondents (53%) expressed that they were comfortable and very comfortable with them. In terms of the integration of basic science with clinical cases and real-life patient scenarios, 52% felt that it was well to very well integrated. When asked about the significance of CBL programs, 98% of respondents rated them as important and very important (Table 3).

**Table 3:** Showing perceptions of faculty members on harmonized competency-based medical curriculum design and structure in Ethiopian public medical schools, 2023

Variables	Response category	Frequency	Percent
Curriculum’s alignment with Intended Learning Outcomes	Not aligned at all	11	12.5
	Not well aligned	25	28.4
	Very well aligned	15	17.0
	Well aligned	37	42.0
	Total	88	100.0
Conduciveness of curriculum’s content and structure to effective teaching and learning	Conducive	53	53.5
	Highly conducive	22	22.2
	Not conducive	12	12.1
	Not conducive at all	12	12.1
	Total	99	100.0
Incorporation of Practical Sessions into the curriculum	Adequately incorporated	15	15.2
	Not adequately incorporated	33	33.3
	Partially incorporated	51	51.5
	Total	99	100.0
Faculty’s perceived comfort with Teaching Methodologies used in the curriculum	Comfortable	28	28.3
	Neutral	15	15.2
	Uncomfortable	32	32.3
	Very comfortable	24	24.2
	Total	99	100.0
integration of basic science with clinical cases and real-life patient scenarios within the curriculum	Neutral	13	13.1
	Not well integrated	34	34.3
	Very well integrated	24	24.2
	Well integrated	28	28.3
	Total	99	100.0
Significance of Community-Based Learning to students’ learning outcomes	Important	49	44.6
	Less important	1	0.9
	Not important	1	0.9
	Very important	59	53.6
	Total	110	100.0

#### Perceived opportunities and challenges with the implementation of the curriculum

This study explored the challenges and opportunities associated with the implementation of a CBMC within public medical schools in Ethiopia. The level of challenges in the curriculum execution was rated by the participants, with 51% perceiving some challenges and 31% considering them to be significant. Conversely, participants recognized overall opportunities or benefits resulting from the new curriculum, which was rated as a significant opportunity by 58% and some opportunities by 24%.

The availability of resources for specific curriculum components, such as rooms for PBL sessions and faculty for preclerkship disciplines, varied among participants. The majority (57%) of respondents found the rooms for PBL sessions inadequate. The availability of full-time faculty for preclerkship disciplines was rated as moderate by 71% of respondents, while 29% rated it as adequate.

The overall availability of faculty training opportunities in innovative teaching approaches was considered adequate by 52% of the participants. Participants rated the availability and utilization of skill labs and simulations, with approximately 72% considering it moderate to sufficient.

Additionally, participant responses highlighted challenges in anatomical dissection sessions, the availability of cadavers and body sourcing, and histological study setups and resources. Only anatomists and academic leaders responded to questions related to anatomy and histological setup (Table 4).

**Table 4:** Showing perceived opportunities and challenges rated by participants in the implementation of the harmonized competency-based medical curriculum in Ethiopian public medical schools, 2023

Variables	Response category	Frequency	Percent
Level of challenges encountered in Implementing the Competency-Based Curriculum	Few challenges	22	18.2
	Significant challenges	37	30.6
	Some challenges	62	51.2
	Total	121	100.0
Existence of opportunities offered by the new curriculum	Few opportunities	22	18.2
	Significant opportunities	70	57.9
	Some opportunities	29	24.0
	Total	121	100.0
Availability of Rooms for Problem-Based Learning (PBL) Sessions	Adequate	11	9.1
	Inadequate	69	57.0
	Moderate	41	33.9
	Total	121	100.0
Availability of full-time faculty to offer courses	Adequately available	24	28.6
	Moderate	60	71.4
	Total	84	100.0
Availability of Faculty Training Opportunities in innovative teaching approaches	Adequately available	57	51.8
	Limited or occasional opportunities	42	38.2
	No training opportunities	11	10.0
	Total	110	100.0
Availability and Utilization of Skill Labs and Simulations	Highly limited	2	2.0
	Insufficient	26	26.3
	Moderate	60	60.6
	Sufficient	11	11.1
	Total	99	100.0
Adequacy and sequence of anatomical dissection sessions	Adequate but with inappropriate sequence	4	6.7
	Inadequate with appropriate sequence	23	38.3
	Inadequate with inappropriate sequence	33	55.0
	Total	60	100.0
Availability of Cadaver and Body Sourcing	Inadequate	15	39.5
	Moderate	11	29.0
	Scarce	12	31.6
	Total	38	100.0
Availability of histological study setups, embryology charts, models, simulations, and similar resources	Adequate	11	18.3
	Inadequate	24	40.0
	Moderate	25	41.7
	Total	60	100.0

### Qualitative insights

The qualitative observations are thematically summarized as follows

#### Curriculum review and modifications

Some medical schools engaged in curriculum review and customizations before and during the implementation of the harmonized curriculum. These modifications encompassed aspects such as sequencing topics, adjusting time allocations, revising content, aligning content systematic approaches, and addressing content ownership issues. Interestingly, few institutions opted for additional reviews after the completion of clerkship years to address real challenges and experiences faced during implementation. Regarding the discussion participants’ opinions, the review and some modifications made to the new curriculum significantly helped them to smoothly implement it in their institution.

#### Hospital visits of preclerkship students

Ideally, hospital visits for preclerkship medical students should commence early in the first preclerkship year as per the curriculum. However, the study revealed variations in the implementation of this practice across schools. The reasons cited for these inconsistencies included the absence of an institution’s own teaching hospital, the fact that general practitioners (GPs) pursuing specialty fellowships have led to physician shortages, and concerns about adding tasks to clinical staff members’ already busy schedules. Consequently, some institutions reduced the frequency of visits, whereas others assigned GPs to facilitate student clinical engagement for each module and maintain the visit.

#### Community visits

The study revealed that student community visits were not consistently implemented in most schools. The challenges cited included resource constraints, such as arranging transport for students to various sites, community fatigue due to frequent visits without addressing community problems, and staff shortages for supervision. However, most of the participants believed that the community visit program was one of the critical aspects of the curriculum.

PBL Implementation: PBL, a key component of the curriculum, was generally well implemented following faculty training in most medical schools. However, challenges included faculty fatigue from repeated PBL tutoring assignments, the lack of dedicated PBL rooms leading to noise disruptions, and the need to search for suitable spaces for each session. Despite these challenges, schools reported the presence of trained tutors, adequate PBL cases and a standardized assessment checklist.

#### Student assessment using OSCE

While OSCE assessments were conducted properly in some schools, a pronounced implementation gap existed in others. This was often attributed to faculty skill and training gaps, limited OSCE station availability, and the absence of standard assessment rubrics.

Whole group sessions: Faculty-facilitated discussion and reflection sessions on the week’s learning activities, a component known as WGS, were rarely implemented in the schools surveyed. Mostly, time constraints due to the curriculum’s busy schedule and the perception of this activity as less significant for some faculty were the primary justifications.

#### Laboratories (Skill Labs)

Most schools reported having been equipped with central laboratories; however, these facilities are commonly shared by different programs, leading to scheduling overlaps. Most participants wished to have separate and dedicated labs for each program.

Common challenges in the implementation of the curriculum: The major challenges reported included faculty lacking reasonable incentives for additional teaching activities, particularly PBL tutoring. This led to demotivated and less committed faculty during the implementation of PBL. Many schools lacked PBL coordinators. Module coordination, although intensive, was often not incentivized, resulting in coordinators with less dedication. Budget and resource constraints pose multifaceted challenges, from procuring lab equipment and standardizing skill lab setups to faculty payments and arranging transportation for community and clinical visits. Some schools also faced staff shortages in certain specializations, complicating curricular activities. Inadequacy of faculty development programs in various aspects of the CBMC and significant truncation of basic science course content in some and adequate course content but a tight schedule to cover topics in most were also important limitations projected by the participants.

#### Opportunities

Participants acknowledged several opportunities brought about by the CBMC. These included students’ early exposure to clinical environments and integration of basic science with clinical cases or real-life case scenarios, enhanced communication skills among students, self-learning and reasoning ability, and problem-solving skills. Moreover, the professional competency development components of the curriculum will support the student in acquiring a practical understanding of how to approach patients and develop the basic skill of examining patients.

## Discussion

The findings of this study shed light on various aspects of the implementation of a harmonized CBMC in public medical schools in Ethiopia.

The overwhelming male majority among the respondents reflects the gender distribution among faculty in the higher educational institutions within Ethiopia [13]. The distribution of experience level, with majority having more than 10 years of higher education, demonstrates a significant level of expertise in their field of specializations among the respondents, which can be valuable for effective curriculum implementation.

This study revealed a mixed landscape regarding the implementation of various aspects of a CBMC. Given the substantial differences between competency-based training programs and traditional curricula in terms of content, design, teaching learning methodology, and assessment, it is crucial not only to familiarize faculty with the new approach but also to provide training on key aspects or innovative elements of the CBMC. The presence of orientation sessions for faculty in some institutions before the curriculum’s commencement is a positive sign, emphasizing the importance of aligning faculty with the curriculum’s goals and teaching and assessment methodologies [14]. However, a significant portion of the faculty who did not attend these sessions warrant attention. Competency-based education thrives on faculty development [3, 14], but the mixed response of the present survey participants to the attendance and adequacy of capacity-building training sessions is indicative of the need for more comprehensive and engaging faculty development programs focused on several aspects of competency-based training.

The initial five-year course schedule of the harmonized modular medical curriculum encompasses a WGS. The variability in implementing WGS in this study may reflect less attention given to the sessions in medical schools. The primary obstacle cited for this variability is the constraint of time due to the tight schedule of the curriculum, which poses a significant challenge. Interestingly, the regularity of PBL activities, a key element of competency-based curricula [15], shows positive traction in the implementation of certain areas of the curriculum.

The review and customization of the newly harmonized CBMC varied, indicating the need for a more uniform approach. The harmonized curriculum is believed to serve as a mother document or reference or menu for schools to adapt to their context based on their educational philosophies, the number and experience of their faculty and the existing infrastructure [Harmonized medical curriculum, 2021]. As the curriculum is new and in the experimentation stage for most medical schools, revision based on implementation encounters should be a continuous process.

Assessment plays a pivotal role in the educational journey, serving not only to verify learning but also to profoundly shape future learning endeavors. In attaining clinical competency, candidates must showcase not only factual knowledge underpinning clinical practice but also the ability to apply this knowledge effectively. OSCE stands out as a common method for assessing various facets of clinical competency, offering a strong means of evaluating practical skills and decision-making abilities in a simulated clinical environment [3]. However, in the present study, the failure to achieve substantial utilization of these methods and tools was mostly due to the inadequacy of OSCE stations, standard assessment rubrics, and faculty training on the utilization of the methods and tools. Therefore, it is highly recommended to pay attention to and avoid the factors that hinder the effective implementation of such a key element of the CBMC.

Regarding CBL, it is noted that the implementation is intermittent or occasional in most schools. Logistical challenges, such as arranging transport for students to various sites, community fatigue due to frequent visits, and staff shortages for supervision, hinder the more consistent implementation of community-based learning among most medical schools in Ethiopia, even though it is mandated by the curriculum as an innovative learning method [Harmonized Medical Curriculum, 2021]. Therefore, medical school leaders need to prioritize addressing these logistical challenges.

Concerning students’ early clinical exposure during preclerkship years, approximately one-third of respondents expressed that students’ clinical exposure during preclerkship years had limitations compared to the standard instructed by the curriculum. Hospital visits are among the most important aspects of competency-based medical education [16, 17]. However, the study revealed variations in the execution of this practice across different medical schools. One significant factor cited in these variations is the absence of an institution’s own teaching hospital. Schools lacking such facilities might face challenges in providing the expected hospital exposure to preclerkship students [18–21]. This brings attention to the infrastructure disparities among institutions. The study also highlights a shortage of physicians available for handling student visits, and concerns about adding tasks to clinical staff’s already busy schedules emerge as a significant barrier. This underscores that staffing challenges can have a cascading effect on the practical aspects of the curriculum.

The faculty’s perceptions and perspectives regarding the curriculum play a pivotal role in gauging the success and effectiveness of educational reforms. In this study, several aspects of faculty perceptions were explored.

Curriculum alignment is a critical factor because it directly influences educational experience and the achievement of predefined learning goals [3, 22]. The findings of this study indicate a notable level of positivity among the faculty regarding the alignment of the curriculum with its intended learning outcomes. This positive perception among a significant portion of the faculty is encouraging, suggesting that a considerable proportion believe that the curriculum is effectively designed to achieve its intended educational objectives. However, it is noteworthy that a substantial portion of the respondents expressed that they did not agree. These findings open avenues for further investigation of the specific aspects of the curriculum that are perceived to require improvement.

The integration of basic science with clinical cases and real-life patient scenarios is a foundational element of competency-based medical education in developing competent and holistic healthcare professionals [23, 24]. The data in the present study suggests the presence of both strengths and areas for improvement in the integration of basic science with clinical scenarios, providing a starting point for targeted investigations.

This study identified a range of challenges and opportunities associated with the implementation of a new CBMC within medical schools in Ethiopia. Challenges include resource constraints, faculty motivation mainly related to faculty incentives, and issues related to curriculum components such as hospital visits, community visits, staff shortages in some specialties, and PBL implementation. These challenges, if not addressed, can compromise the overall effectiveness of the curriculum’s implementation. On the positive side, large proportion of respondents recognized that the curriculum offered significant opportunities, including early exposure to clinical environments, enhanced communication skills, and the development of self-learning abilities and problem-solving skills. Leveraging these skills can contribute to the holistic development of medical students and help them prepare for the dynamic challenges of healthcare practice [3, 25].

Participants’ responses on incorporating practical sessions, skill lab availability, and simulation use suggest a need for improvement to ensure effective implementation of hands-on learning aligned with the competency-based approach.

A considerable portion of respondents perceived anatomical dissection sessions as inadequate with an inappropriate sequence. This indicates a critical gap in the practical application of theoretical knowledge. Challenges in terms of cadaver availability highlight a significant impediment to effective anatomical education. The cadaver scarcity may hinder students’ hands-on learning experiences [26]. Addressing this challenge requires a comprehensive strategy, potentially involving strengthening collaborations with external sources or anatomical donations and initiating additional cadaver preparation or embalming centers.

## Conclusion

The mixed landscape in the implementation of the harmonized competency-based medical curriculum reveals both positive trends and areas for improvement. Faculty perceptions play a pivotal role, and the positive outlook on the alignment of curriculum design with intended learning outcomes and conducive content structure is promising. However, challenges related to practical sessions, assessment methodologies, WGS, and community-based learning indicate areas for refinement. Resource constraints emerge as a significant challenge. Overall, this study provides valuable insights into the complexities of implementing a competency-based medical curriculum and highlights the need for ongoing evaluation and fine-tuning to ensure its success.

### Limitations of the study

The study focused on faculty perspectives, but student perspectives are also crucial for understanding the quality of medical education. In addition, private medical schools were not included in this study. Future research could incorporate student feedback and private medical schools.

## Data Availability

The datasets used and/or analysed during the current study are available from the corresponding author on reasonable request.
